# Hypertrophic Osteoarthropathy: A Case of an Undifferentiated Polyarthritis

**DOI:** 10.7759/cureus.51145

**Published:** 2023-12-27

**Authors:** Margarida M Carvalho, Sara Raimundo

**Affiliations:** 1 Pulmonology Department, Centro Hospitalar Trás-os-Montes e Alto Douro, Vila Real, PRT

**Keywords:** periostitis, polyarthritis, lung adenocarcinoma, paraneoplastic rheumatic disease, hypertrophic osteoarthropathy

## Abstract

Hypertrophic osteoarthropathy is a paraneoplastic syndrome and is considered an important secondary cause of rheumatic disease. It typically manifests as tibial and femoral bone pain, with arthralgia or synovitis of adjacent joints also being common findings. Usually, musculoskeletal symptoms accompany the course of the disease, disappearing with treatment of the neoplasm and recurring coincidentally with the tumor relapse. The authors report a case of a patient with hypertrophic osteoarthropathy, whose etiological study allowed the diagnosis of a lung adenocarcinoma, particularly challenging due to the patient's young age and the absence of associated symptoms.

## Introduction

Hypertrophic osteoarthropathy (HOA) is a rare paraneoplastic disease that can be extremely debilitating [1]. The primary form of HOA is not associated with any other medical condition [2]. The secondary form, previously referred to as hypertrophic pulmonary osteoarthropathy, is usually associated with primary lung malignancies, particularly non-small cell lung carcinoma [2,3]. In fact, periosteal reaction involving long bones, without history of trauma or an underlying bone lesion, should lead to prompt investigation of secondary causes [3].

HOA is characterized by the clinical triad of oligoarthritis or polyarthritis, clubbing of the fingers, and periostitis of the distal ends of long bones [2,4-6]. Arthritis is the most frequent feature and affects most predominantly the knees, ankles, elbows, wrists, and metacarpophalangeal (MCP) and proximal interphalangeal (PIP) joints [7,8]. It is often symmetrical, painful, and associated with tenderness of the adjacent bones [5-9]. There are no useful blood tests for HOA diagnosis, but the erythrocyte sedimentation rate (ESR) is usually increased [6,8]. Alkaline phosphatase can be increased in patients with widespread periostitis [8].

Some factors should raise suspicion of paraneoplastic syndromes, namely: personal or family history of neoplasia, exposure to carcinogens, constitutional symptoms, age over 50 years at diagnosis, and disease refractory to treatment [10]. Clinically, the musculoskeletal symptoms accompany the course of the disease [2,8]. Thus, the symptoms disappear with the treatment of the neoplasm, and their recurrence coincides with tumor relapse [2,8]. In this case report, we describe a representative case of a young patient with musculoskeletal symptoms suggestive of a rheumatic disease which was later found to be a paraneoplastic syndrome.

This article was previously presented as a meeting poster at the XXX Congresso de Pneumologia do Norte/XXXVI Jornadas Galaico Durienses on March 10, 2023.

## Case presentation

**Figure 1:**
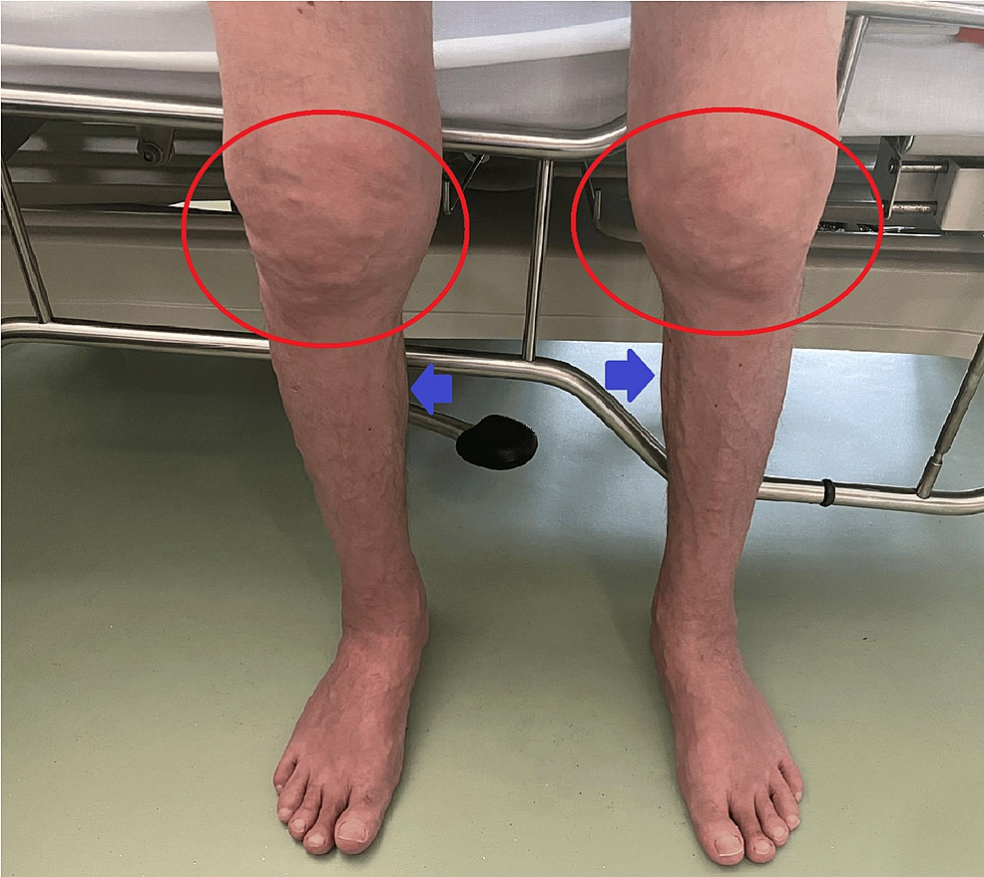
Joint swelling of the knees, with some flexion (red circles); atrophy of the gastrocnemius (blue arrows).

We report the case of a 44-year-old male, with no significant medical history. He was a former smoker with a smoking history of 21 pack-years. The patient was referred to a rheumatology consultation due to arthralgia in the upper and lower limbs. On the clinical examination, he presented with pain on passive and active mobilization of the knees, arthritis in the hands, knees, and ankles, and synovitis of small joints (Figure 1). He had no palpable lymph nodes.

**Figure 2:**
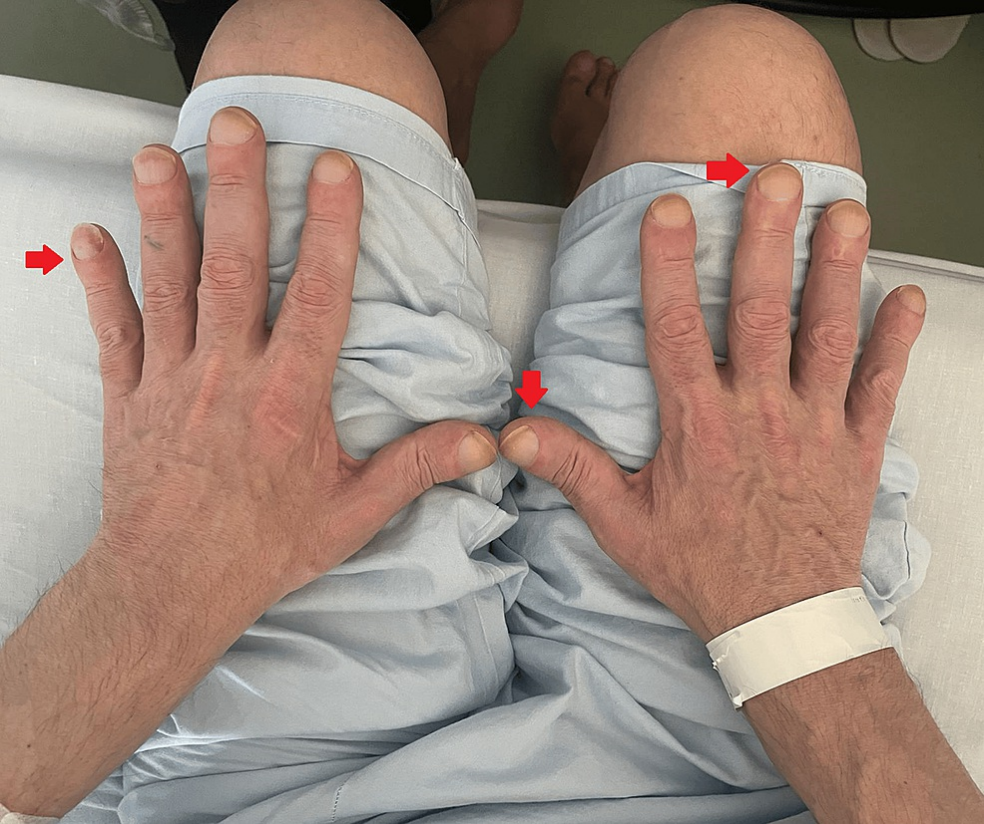
Digital clubbing (red arrows).

Blood test analysis revealed slight anemia (hemoglobin 11.2g/dL, reference range (RR) 13.5-17.5 g/dL) and elevation of inflammatory parameters such as leukocytosis of 19.2/L (RR 4.5-11.0/L), C-reactive protein (CRP) of 4.20 mg/dL (RR <0.3mg/dL), and ESR of 60 mm/hr (RR ≤15 mm/hr). Kidney, liver, and thyroid function values were normal. Immunology showed a positive antinuclear antibody of 1:160 titer (RR <1:60), homogeneous pattern. Rheumatoid factor, anti-cyclic citrullinated peptide antibody, and HLAb27 were negative. Serologies for hepatitis B and C, human immunodeficiency virus (HIV), and cytomegalovirus (CMV) were also negative. Thus, undifferentiated polyarthritis was assumed. During follow-up, the patient presented with digital clubbing (Figure 2). At this point, the patient denied any respiratory and constitutional symptoms.

**Figure 3:**
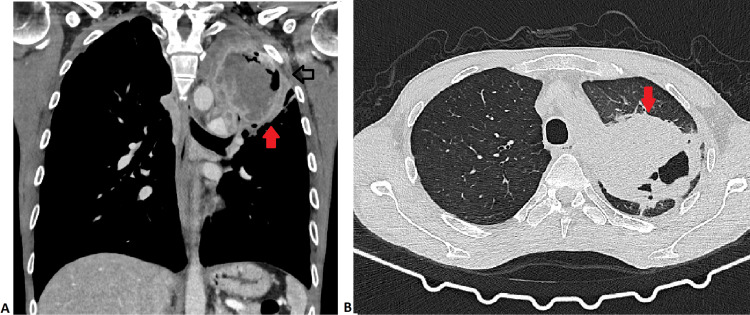
Chest CT scan: (A) coronal, mediastinal window; and (B) axial, lung window. Mass of heterogeneous density in the left upper lobe, predominantly necrotic, with heterogeneous peripheral enhancement and presence of cavitation (red arrow). CT: computed tomography

This signal led to the performance of a chest computed tomography (CT) (Figure 3A and Figure 3B) that revealed a mass of heterogeneous density in the left upper lobe, measuring approximately 9.5 x 8.5 x 8 cm, predominantly necrotic, with heterogeneous peripheral enhancement and presence of cavitation. It had an extensive contact surface with the pleura in the region of the pulmonary and mediastinal apex, with multiple peripheral satellite micronodules. Furthermore, there were a 4 mm cavitated nodule in the basal lateral segment of the right lower lobe and multiple adenopathies in the left lung hilum and in the aortic-pulmonary window. 

A suspicion of primary lung cancer was raised, and the patient was referred to the oncological pulmonology consultation where he continued the study. A bronchofibroscopy was performed revealing decreased lumen of the apicoposterior bronchus. Bronchial biopsies and brushing were performed for cytological study, both negative for neoplastic cells. The histology of transbronchial lung biopsy revealed mucosal fragments with infiltration by non-small cell carcinoma, solid pattern, and areas of necrosis, whose immunohistochemistry study showed a positive thyroid transcription factor-1 (TTF1) and negative p40, favoring primary lung adenocarcinoma as final diagnosis.

A positron emission tomography (PET)/CT was performed, which showed a massive left pulmonary hypermetabolic lesion, involving the parenchyma, hilum, and aortopulmonary window. In the same lung, several densifications were observed, some grossly nodular and others more diffuse, with high metabolism. In the right lung, increased uptake was observed in several diffuse pulmonary densifications, with moderate metabolism.

**Figure 4:**
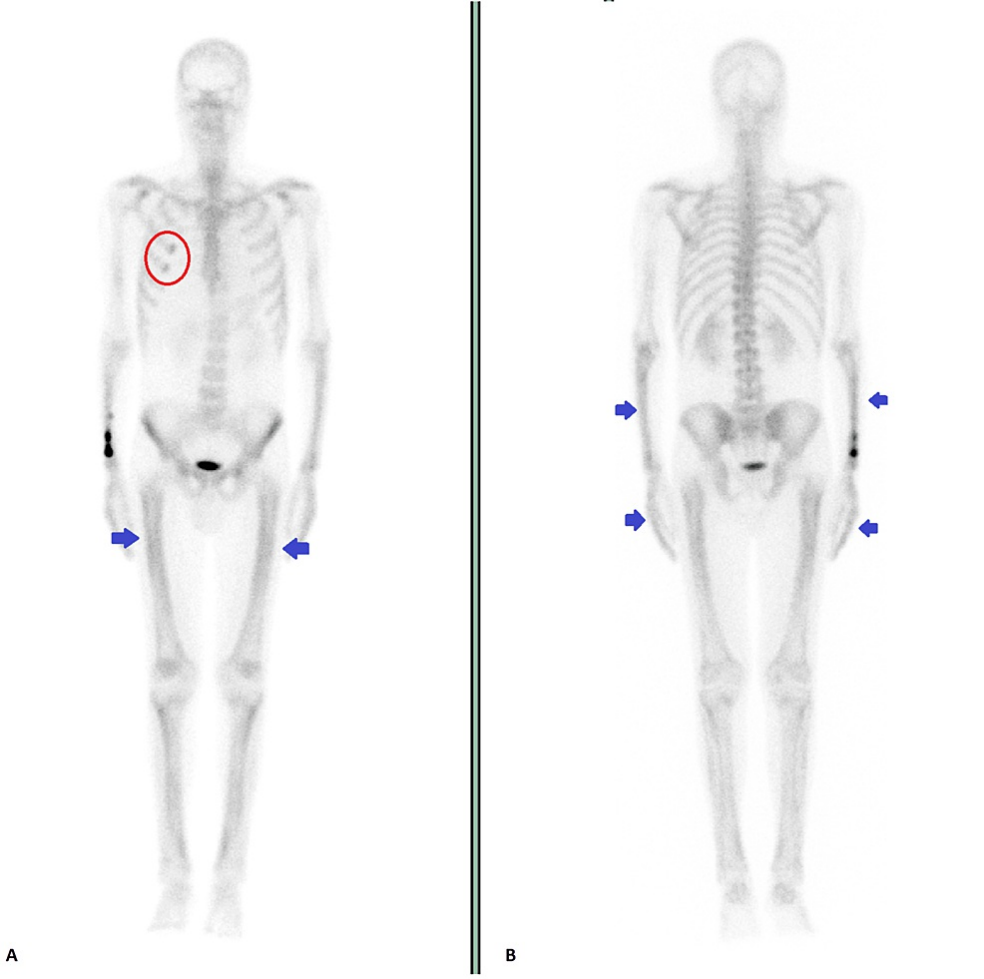
Bone scan: (A) full body, anterior view; and (B) full body, posterior view. Foci of hyperfixation of the radiopharmaceutical in the anterior ends of the third and fourth right costal arches (red circle), probably related to post-traumatic pathology. Marked and diffuse hyperfixation of the long bones on the lower limbs and upper limbs and also at the level of the hands, in an aspect suggestive of hypertrophic osteoarthropathy (blue arrows).

Magnetic resonance imaging (MRI) of the brain revealed one metastatic intra-axial lesion with left anterior temporal cortical topography, with 15.5 mm of longest axis and with moderate edema halo, conditioning regional sulcal effacement. The patient realized a bone scan (Figure 4A and Figure 4B) which did not demonstrate suspicious images for bone metastases of the osteoblastic type. It showed marked and diffuse hyperfixation of the long bones of the lower and upper limbs and also at the level of the hands, in an aspect suggestive of HOA.

The malignancy was staged as T4N3M1b-IVA, according to TNM 8th edition staging, with contralateral lung metastasis and a single brain metastatic lesion. No target mutations were present, and programmed death-ligand 1 (PD-L1) expression was less than 1%. The patient had an Eastern Cooperative Oncology Group (ECOG) performance status of 0. He then received stereotactic radiotherapy to the brain lesion and was started on chemotherapy with carboplatin, pemetrexed, and pembrolizumab for four cycles, resulting in a partial response, followed by eight cycles of maintenance. His musculoskeletal symptoms regressed completely with cancer-directed therapy.

Two months later, the patient had exuberant swelling of the tibiotarsal joints and stiffness of the knees, with associated arthralgia. The chest CT scan revealed volumetric increase and greater heterogeneity of solid and perifissural cavitated lesion in the apicoposterior segment of the left upper lobe, with Response Evaluation Criteria in Solid Tumours (RECIST) criteria of progressive disease. The patient started second-line treatment with docetaxel and nintedanib, with significant improvement of the joints' swelling and stiffness and resolution of arthralgia.

One month after finishing the sixth cycle of docetaxel, there was a new worsening of joint swelling, in the knees, ankles, and hands, with associated joint tenderness. The chest CT scan confirmed local disease progression, and the patient initiated third-line chemotherapy with gemcitabine. A rebiopsy of the lesion was performed for molecular study, which confirmed the absence of mutations with available targeted therapy. There was also disease progression in the central nervous system, with the appearance of one brain metastasis, which led to significant clinical deterioration due to intracranial hypertension and prevented the patient from receiving surgical treatment. In order to relieve the symptoms of intracranial hypertension, a ventriculoperitoneal shunt was placed, and the patient was proposed for best supportive care. The patient died five months later.

## Discussion

This clinical case highlights the challenges associated with diagnosing neoplasms in young and asymptomatic patients, especially when rheumatological symptoms obscure the clinical presentation. Despite HOA being characterized by arthritis, periostitis in the distal ends of long bones, and finger clubbing, these symptoms were mistakenly interpreted as a primary rheumatic disease. This may be attributed to the fact that the patient did not exhibit respiratory or constitutional symptoms and probably due to his young age. The association between rheumatic diseases and malignancy is very complex [4].

Although some patients with HOA can be asymptomatic, most patients, in particular those with malignant lung tumors, have periostosis of tubular bones which can be evident in areas that are not covered by muscles, such as the ankles and wrists [2,6]. Usually, it is accompanied by pain of the involved area, which can be incapacitating, typically at the tibial and femoral bones [2,9]. Arthropathy or synovitis of adjacent joints are common [7,9]. Arthropathy can vary from mild arthralgia to a diffuse polyarthritis [7]. The arthritis is often symmetrical and affects the knees, ankles, elbows, wrists, and MCP and PIP joints [7,8]. It can be associated with tenderness of the adjacent bones, with decreased range of motion of the affected joints [6,8].

Other characteristic of patients with HOA is clubbing of fingers and toes which can be the only manifestation of the syndrome [6]. Digital clubbing is characterized by periungual edema, softening of the nail bed, and increase in the angle of the hyponychium [6]. It can be accompanying with a burning pain in the tips of fingers and toes [8].

Radiographs of the extremities may show symmetrical and bilateral diaphyseal subperiosteal proliferation, particularly of tibia, fibula, ulna, metacarpal, and metatarsal bones [5,6,9]. It can be seen as linear ossification separated by a radiolucent zone from the underlying cortex [5,8]. Bone scintigraphy typically demonstrates a symmetric linear increased uptake along the diaphyseal and metaphyseal surfaces of long bones, before periosteal new bone formation is apparent on radiography, allowing an early diagnosis [2,7]. Bone scintigraphy can also be used to evaluate the response to therapy [2]. PET can also detect the highly inflammatory pattern of periostitis [9].

For patients with painful osteoarthropathy, nonsteroidal anti-inflammatory drugs (NSAIDs) are effective in most cases, relieving symptoms [2,6]. The complete remission of the underlying cancer, by either resection, radiotherapy, or chemotherapy, often leads to rapid remission of the arthritis and diminishing of the digital clubbing [2,8]. Without treatment of the cause of HOA, symptoms often persist with bone pain, recurrent synovitis, and painful joint effusions [8].

## Conclusions

In this case, the patient had a paraneoplastic rheumatic syndrome with musculoskeletal symptoms, which etiological study allowed the diagnosis of a lung adenocarcinoma, particularly challenging in this patient due to his young age and the absence of associated symptoms. It also reinforces the importance of considering rheumatological paraneoplastic syndromes, especially in patients without the immunologic markers characteristic of rheumatic diseases. Prompt identification of these signs can guide the investigation towards possible neoplasms, playing a crucial role in the timely diagnosis and proper management of neoplasms in their early stages. It should be noted that, in these cases, the arthritis can improve with NSAIDs, but its resolution depends on the treatment of the underlying neoplastic disease, with recurrence and worsening of symptoms with tumor recurrence.
